# A Rare Presentation of Quadrigeminal Cistern (Tectal Plate) Lipoma With Visual Hallucinations in a Patient of Schizophrenia: A Case Report

**DOI:** 10.7759/cureus.31132

**Published:** 2022-11-05

**Authors:** Vaishali Sehgal, Pradeep S Patil, Surabhi Mitra

**Affiliations:** 1 Psychiatry, Jawaharlal Nehru Medical College, Datta Meghe Institute of Medical Sciences, Wardha, IND

**Keywords:** functional psychosis, tectal plate, visual hallucinations, schizophrenia, quadrigeminal cistern, intracranial lipoma

## Abstract

Intracranial lipomas are rare benign congenital tumours, consisting of <0.1% of all primary brain tumours. They are asymptomatic and are thus incidentally discovered whenever brain imaging is advised due to any other organic cause. These lipomas can rarely present with acute psychosis or schizophrenia-like symptoms characterised by abnormal perceptions, cognition, mood, and behaviour. One of the most frequent symptoms of schizophrenia is hallucination. In patients with schizophrenia, hallucinatory experiences can have an impact on any of the five senses; however, auditory hallucinations are the most common. Although tactile, olfactory, and gustatory hallucinations are uncommon, visual hallucinations are also frequently experienced; their existence should prompt a clinician to think about whether there may be an underlying medical or neurological condition that is the root of the entire syndrome. Thus, the presence of visual hallucinations in a patient with psychosis should not be neglected and brain imaging should be done to rule out any organic brain disease. Schizophrenia-like symptoms in a patient of quadrigeminal lipoma are rare and not many cases have been reported in this context. Quadrigeminal lipoma presenting with headache, seizure, loss of consciousness, and aggression has been reported but not with acute psychosis or schizophrenia. Hereby, we report a case of a 35-year-old female who presented with psychosis with predominant visual hallucinations, which suspected an underlying organic pathology and brain imaging showed the presence of quadrigeminal cistern lipoma. The idea behind choosing this case is to report a very rare condition with an even more atypical associated presentation.

## Introduction

Intracranial lipomas are rare benign congenital tumours, consisting of <0.1% of all primary brain tumours [1]. They are asymptomatic and are thus incidentally discovered whenever brain imaging is advised due to any other organic cause. The abnormal differentiation and abnormal persistence of meninx primitiva (subarachnoid precursor), accompanying parenchymal anomalies, and traversing structures of intracranial lipomas constitute the most plausible and acceptable theory of how these lipomas develop [2].

Types of intracranial lipomas based on their location and occurrence are the following: 1. Pericallosal lipoma (45%) - In 50% of instances, this is accompanied by corpus callous agenesis. 2. Quadrigeminal cistern lipoma (25%) - It is related to inferior colliculus underdevelopment. 3. Suprasellar cistern lipoma (15%). 4. Cerebellopontine angle lipoma (10%) - This type of lipoma affects the facial nerve and vestibulocochlear nerve. 5. Sylvia fissure (5%). 6. Choroid plexus lipoma (rare). 

Since they are typically asymptomatic, no specific treatment is necessary. Resection attempts have really resulted in a high rate of morbidity with very little benefit. Only if they exhibit additional abnormalities, such as seizures or hydrocephalus, treatment is recommended. Due to the extremely vascular nature of the lipomas and their strong adherence to the surrounding tissues, radical surgical incision is typically not advised as it may cause a high rate of morbidity and mortality [2].

These lipomas can rarely present with acute psychosis or schizophrenia-like symptoms characterised by abnormal perceptions, cognition, mood, and behaviour. The National Institute of Mental Health places the lifetime prevalence of schizophrenia between 0.6% and 1.9%. Schizophrenia strikes men and women equally. Diseases have different presentations and progressions in males and females. Males typically experience the onset before females. Men often experience onset between 10 to 25 years while women typically experience it between 25 to 35 years, with a second peak developing in the middle years. The Diagnostic and Statistical Manual of Mental Disorders, Fifth Edition, (DSM-5) identifies the primary clinical symptoms of schizophrenia as delusions, hallucinations, disorganised speech, grossly disorganised or catatonic behaviour, and negative symptoms such as a diminished emotional expression or avolition. One of the most frequent symptoms of schizophrenia is hallucination. In patients with schizophrenia, hallucinatory experiences can have an impact on any of the five senses. However, auditory hallucinations, frequently featuring frightening, offensive, accusing, or insulting voices, are the most typical type of hallucination. A voice may talk to another voice or voices, or it may make comments on the patient's life or behaviour [3].

Although tactile, olfactory, and gustatory hallucinations are uncommon, visual hallucinations are also frequently experienced; their existence should prompt a clinician to think about whether there may be an underlying medical or neurological condition that is the root of the entire syndrome. Therefore, the presence of visual hallucinations in a patient with psychosis should not be neglected and brain imaging should be done to rule out any organic brain disease. Hereby, we report a case of a 35-year-old female who presented with psychosis with predominant visual hallucinations, which suspected an underlying organic pathology and brain imaging showed the presence of quadrigeminal cistern lipoma.

This article was previously presented as a paper abstract at the 73rd Annual National Conference of Indian Psychiatric Society (ANCIPS) on March 25, 2022.

## Case presentation

The indexed case is a 35-year-old married female, educated till 11th standard, housewife by occupation, belonging to a low socioeconomic background, with well-adjusted pre-morbid personality, without any significant past history of psychiatric illness, having a family history of psychiatric illness, i.e., depressive disorder in elder brother and schizophrenia in younger sister, who presented with symptoms of sleep disturbance, muttering to self, smiling to self, with aggressiveness, having suspiciousness towards family members, and with auditory and visual hallucinations since last 1.5 year. 

Her symptoms gradually started 1.5 years ago when she started having difficulty in initiating and maintaining sleep. She used to go to bed at ten pm and was unable to sleep by one am and used to wake up in the middle of the night and start wandering in the house. 

The patient started showing suspicious behaviour toward her mother-in-law. She claimed that she gives her poisonous food. The patient denied taking food offered by her and also did not let her children consume the food given by her. She was also suspicious towards her sister-in-law that she steals her personal belongings and started blaming her for anything she could not find in the house. The patient’s daughter reported that she used to get irritable on trivial issues and used to get physically aggressive toward her.

The patient also started exhibiting hallucinatory behaviour in the form of muttering and smiling to herself. She had visual and auditory hallucinations as she claimed that she heard voices and was also able to see both of her brothers who died a few years ago. There was no prior history of fearfulness, low mood, decreased interaction with family members, death wishes and suicidal ideation, over-talkativeness, or grandiose talks. 

Her symptoms had gradually increased in the past five months. There was no psychiatric consultation taken for the same. However, they visited multiple faith healers and Ayurvedic practitioners. No improvement in the symptoms was reported. 

The patient's past history revealed the presence of chronic suppurative otitis media (CSOM) in both ears in childhood, and thus, she has associated hearing loss in both her ears since 20 years ago. No surgical intervention was done for the same but the patient had been using a hearing aid in the left ear since then. There was no history suggestive of seizure disorder, delirium, substance intoxication, bipolar illness, dissociative disorders, or any other disorders. 

On physical examination, heart rate (HR) was 74/min, blood pressure (BP) was 110/70 mm of Hg, oxygen saturation (SpO^2^) was 99% on room air, BMI was 24 kg/m^2 ^(within normal limits), there were no signs of pallor, icterus, clubbing, cyanosis, lymphadenopathy and oedema, and no abnormality was detected on systemic examination.

On mental status examination, consciousness and orientation were found to be intact, and she was moderately built, adequately dressed, kempt and tidy. She maintained touch with the surroundings, eye-to-eye contact with the examiner was initiated but not maintained, her attitude towards the examiner was cooperative, rapport was established with difficulty, and psychomotor activity was normal. Her speech was decreased in terms of intensity, pitch, and quality, was relevant and goal-directed, and her reaction time was quite increased. Her affect was blunt. Delusion of reference and persecution was present. Second-person auditory hallucinations and visual hallucinations were present. 

On examining cognitive functions, attention and concentration were aroused but ill-sustained, memory was intact, and abstract ability and judgement were impaired. 

Course during hospital stay

**Figure 1:**
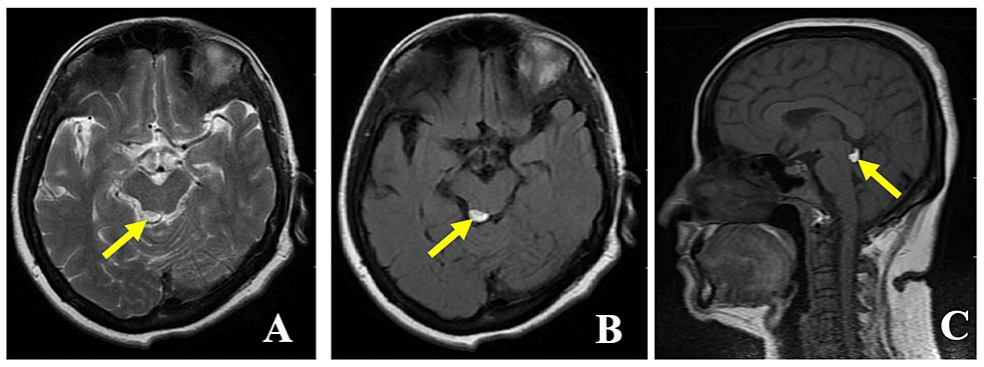
Non-contrast MRI brain images: A) T2 axial section showing a lipoma in the quadrigeminal cistern, B) Fluid-attenuated inversion recovery (FLAIR) axial section showing a lipoma in the quadrigeminal cistern, C) T1 Sagittal section showing a lipoma in the quadrigeminal cistern

The patient was diagnosed with schizophrenia as per the International Classification of Diseases (ICD)-10th revision criteria. Her routine blood investigations were ordered, which were within normal limits. She was started on tablet olanzapine 5 mg which was gradually increased to 20 mg. ENT opinion was taken in view of bilateral hearing loss. An audiogram showed profound hearing loss in the right ear and severe mixed hearing loss in the left ear. Hearing aids were advised for both ears. MRI brain plain was ordered in view of visual hallucinations, which showed a well-defined T1 and fluid-attenuated inversion recovery (FLAIR) hyperintense lesion measuring 10 x 6 mm in size seen in the region of the quadrigeminal cistern (tectal plate), features suggestive of tectal plate lipoma. Gadolinium was not used as a contrast medium in this case. The non-contrast MRI images of the patient are shown in Figure 1. 

## Discussion

The patient presented with typical schizophrenia-like symptoms with predominant visual hallucinations, which suspected an underlying organic pathology which prompted a radiological investigation and neurological reference. MRI brain pinpointed the tumour’s location to the quadrigeminal cistern (tectal plate). Quadrigeminal cistern lipomas in themselves are very rare tumours, accounting for 25% of all intracranial lipomas. Among these, tectal plate lipoma presenting with psychosis is a very rare entity and till now only one such case has been reported [1]. These rare developing tumours form when primitive meninges persist and grow abnormally, but they are very slow-growing tumours and rarely cause symptoms, and thus, never necessitate surgical removal. About 20% of individuals will experience mass consequences from these lipomas, which may result in cognitive deficits, elevated intracranial pressure, and obstructive hydrocephalus. Radical surgical incision of these lipomas is usually contraindicated as it may result in high morbidity and mortality due to the highly vascular nature of the lipomas and also the strong adhesion to the surrounding tissues [4]. Investigations preferred for these lipomas are radiological investigations only. Histopathological confirmation of a diagnosis of lipoma has traditionally not been pursued in practice because prior research had suggested that doing so was unnecessary. Lipomas are benign tumours that have been hypothesised to encapsulate nerves and arteries of adjacent structures, resulting in a wide range of symptoms. A review of literature showed that headache, loss of consciousness, seizures, cranial nerve palsy, abnormal behaviour, and aggression were just some of the symptoms of these quadrigeminal lipomas, but psychosis has not been reported much [5]. 

Thus with the help of this medium, we have reported a case of tectal plate lipoma presenting with symptoms of schizophrenia. Due to the absence of brainstem compression symptoms and the fact that operating near the brainstem is, notoriously, challenging due to the risk of injury to the brainstem itself and surrounding tissues, surgery was not indicated. Moreover, the patient responded well to antipsychotics, leading our team to believe that the psychiatric symptoms were not caused by the tumour. The patient’s caregiver reported improvement in overall symptoms including visual hallucinations over a period of 2-4 weeks. Similar lipomas have been linked to headaches, unconsciousness, and seizures in the past, all of which improved after medical intervention [6]. 

## Conclusions

In conclusion, it should be kept in mind that the presence of visual hallucinations in a patient with psychosis should not be neglected and prompt brain imaging should be done to rule out any organic brain disease. The varied presentations of organic brain pathology may help in early detection, better management, and assessing prognosis and outcome. Specific diagnosis with help of radiological investigation is necessary as most chronic organic brain disorders cannot be reversed leading to poor outcome and prognosis. 
